# Posterior Circulation Stroke in Patients With Dizziness: Diagnostic Challenges and Practical Considerations for Emergency Care

**DOI:** 10.1016/j.acepjo.2026.100532

**Published:** 2026-10-21

**Authors:** Andrea L. Klein, Allison N. Seifert, Jonathan A. Edlow, Andrew B. Moore, Opeolu Adeoye, Laura Heitsch, Tracy E. Madsen, Katherine D. Mayes

**Affiliations:** 1 Department of Emergency Medicine, Virginia Tech Carilion School of Medicine, Roanoke, Virginia, USA; 2 Virginia Tech Carilion School of Medicine, Roanoke, Virginia, USA; 3 Department of Emergency Medicine, Beth Israel Deaconess Medical Center, Harvard Medical School, Boston, Massachusetts, USA; 4 Department of Health Systems and Implementation Sciences, Virginia Tech Carilion School of Medicine, Roanoke, Virginia, USA; 5 Department of Emergency Medicine, Washington University School of Medicine, St. Louis, Missouri, USA; 6 Department of Emergency Medicine, College of Medicine, University of Vermont, Burlington, Vermont, USA

## 1 Background

Posterior circulation stroke (PCS) accounts for approximately 20 to 30% of ischemic strokes, with an estimated annual incidence of 1.17 million.^1^^,^^2^ Diagnosis can be challenging because posterior circulation ischemia may present with common, usually benign symptoms such as dizziness, vertigo, headache, nausea, or vomiting. However, more localizing findings, including sudden dysphagia, diplopia, dysarthria, or ataxia, should not be considered nonspecific and warrant a careful neurologic examination directed at posterior circulation structures. Population-based data suggest that stroke is uncommon among emergency department (ED) patients presenting with dizziness, but PCS may be missed when subtle focal findings are absent or not elicited.^3^

Most prehospital and ED stroke screening protocols use Face, Arms, Speech and Time (FAST), or Balance, Eyes, Face, Arms, Speech and Time (BE-FAST)-based assessments to identify possible stroke, whereas the National Institutes of Health (NIH) Stroke Scale (NIHSS) is used primarily to quantify neurologic deficit severity and support treatment decisions after stroke is suspected. Both approaches may underemphasize posterior circulation findings. Though posterior examination maneuvers have been shown to improve sensitivity of PCS detection,^4^ no universally accepted supplemental scale exists for PCS. Evidence for time-dependent reperfusion in PCS is less robust than for anterior circulation stroke and is strongest for basilar artery occlusion; nevertheless, timely recognition remains important to facilitate appropriate imaging to identify the underlying vascular mechanism, specialist consultation, consideration of reperfusion therapy in eligible patients, and to begin secondary stroke prophylaxis treatments.^5^ This review examines the strengths and limitations of available severity scales, diagnostic tools, and imaging strategies for patients with dizziness or vertigo and offers practical recommendations for their use in emergency care.

## 2 Clinical Evaluation

The NIHSS omits important signs of PCS, including truncal ataxia, nystagmus, and gait imbalance.^6^^,^^7^ Historically, a NIHSS ≤5 has been considered mild stroke^8^ although low scores can still accompany clinically important disability. In one large international cohort, the mean NIHSS score among PCS patients was 3.8, despite a 39.5% rate of death or dependency at discharge.^9^ The NIHSS, therefore, remains useful for documenting severity and treatment eligibility after stroke is suspected, but it should not be used alone to exclude PCS. In dizzy patients with a low NIHSS, clinicians should assess vascular risk factors and focus the examination on nystagmus, gait, and truncal stability.

### 2.1 Tools Designed to Measure Stroke Severity

Several posterior stroke–specific severity scales have been developed to improve characterization of deficits after PCS has already been suspected or diagnosed (Table). The Israeli Vertebrobasilar Stroke Scale (IVBSS) demonstrated reproducibility and validity in cohorts with known PCS, although broader testing in undifferentiated ED populations is still needed.^12^ The expanded NIHSS (e-NIHSS) retains all NIHSS components with expanded eye, facial, and ataxia scoring; scores ≥8 showed 82% sensitivity and 81% specificity for adverse outcomes at 90 days in patients with PCS.^14^ The Posterior NIHSS (POST-NIHSS) incorporates gait/truncal ataxia, dysphagia, and abnormal cough and demonstrated greater prognostic accuracy for poor outcomes than the NIHSS.^15^ The Adams Scale of Posterior Stroke (ASPOS) has also been evaluated, with scores >3 associated with PCS in a single-center cohort.^16^ These instruments may improve severity or prognostic assessment in selected patients, but current evidence does not support their routine use as diagnostic screening tools in the ED.

**Table:** Tools designed to measure stroke severity and diagnose PCS.

Tool name	Original cohort	Original intention	Components of tool	Secondary validation? (Yes/No, explanation)	Findings of study	Utility for use in the ED
Tools designed to measure stroke severity						
NIHSS^6^^,^^10^	Pilot studies of patients receiving tPA for ischemic stroke/ study of NIHSS reliability in 162 investigators	To assess stroke severity in trials of reperfusion therapy	Comprised 11 components pertaining to level of consciousness, facial palsy, vision, neglect, motor function, sensation, language, speech, limb ataxia	No: not validated for posterior stroke severity	N/A	Useful for severity assessment and treatment eligibility after stroke is suspected; not a diagnostic tool and may underestimate PCS. Consider gait and truncal testing when the score is low.
IVBSS^11^	Prospective cohort study of 43 PCS patients in 3 Israeli hospitals	To assess severity in known PCS	Select components of the NIHSS (LOC, Gaze, Motor, Ataxia, Sensory, Dysarthria, Sensory) + Diplopia, Dysphagia and Gait	Yes: assessed the reliability, validity and psychometric properties of a Polish version in 126 known PCS patients^12^	Replicated high reproducibility, repeatability and accuracy of the IVBSS to assess stroke severity	Validated only in patients with known PCS; not recommended to screen undifferentiated ED patients.
e-NIHSS^13^	Prospective single-center cohort study of ‘suspected’ PCS (n = 22) or ACS (n = 25) patients in Italy	Compared e-NIHSS and NIHSS scoring for severity of ACS vs PCS	Retains all the NIHSS components with expanded eye, facial palsy and ataxia scoring	Yes: compared ability of the e-NIHSS to NIHSS in confirmed PCS to predict 90-day mRS ≥3^14^	e-NIHSS was better for predicting 90-day outcomes (ROC analysis showed 82% sensitivity and 81% specificity for e-NIHSS ≥8)	Assesses severity and prognosis; not validated as a diagnostic screen in undifferentiated ED patients.
POST-NIHSS^15^	Retrospective analysis of 202 PCS patients with NIHSS < 10 in the BATMAN registry	Develop a tool to improve prognostic accuracy for PCS with mild-to-moderate symptoms	Derivation cohort determined POST-NIHSS should include additional points for gait/truncal ataxia, dysphagia, and abnormal cough to regular NIHSS scoring	Yes: applied the POST-NIHSS tool to 65 prospectively recruited PCS patients in the BATMAN registry to assess prognostic ability for poor outcome (mRS ≥3) at 90 days	POST-NIHSS showed significantly higher prognostic accuracy for worse outcome than NIHSS in both the validation cohort as well as combined validation + derivation cohorts	Derived and validated in known PCS for prognostication; not validated to identify PCS in an undifferentiated ED population.
ASPOS^16^	Prospective single-center cohort study of 126 PCS patients in Poland	Designed to assess severity in known PCS	Select components of the NIHSS (LOC, Motor, Ataxia, Sensory) + expanded eye and pharynx exams as well as balance testing	No: ∗tested the efficacy of ASPOS to predict PCS in the ED for an Indian population (not validated for original purpose)^17^	A score > 3 was significantly correlated with PCS. ROC analysis shows ASPOS scores have 63% sensitivity and 95.7% specificity for predicting PCS	Single-center evidence with limited external validation; insufficient evidence for routine diagnostic use.
Tools to diagnose PCS using history and clinical features						
TriAGe+^18^	Observational cohort of 498 patients in Japan presenting to the ED with dizziness or vertigo	To detect stroke in patients with dizziness or vertigo	8 components: no triggers, atrial fibrillation, male biologic sex, blood pressure, brainstem or cerebellar dysfunction, clinical features (motor or speech), dizziness (vs. vertigo), no history of vertigo/dizziness/ labyrinth/ vestibular disease	Yes: validated in single-center prospective observational study of 357 patients with dizziness or vertigo^19^	For TriAGe+ ≥ 7, sensitivity was 81.0%, specificity was 77.6%. AUC = 0.88	Single-center validation with no robust multicenter head-to-head comparisons; investigational adjunct rather than a standalone ED tool.
Sudbury Vertigo Score^20^	Prospective cohort study of 2078 patients in 3 EDs in Canadian teaching hospitals	To predict serious diagnoses in patients with vertigo	Stroke risk factors (male biologic sex, age > 65 years, history of diabetes, history of hypertension), neurological deficits (motor/sensory, cerebellar), BPPV diagnosis	Yes: validated in separate retrospective cohort of 4559 ED patients with vertigo, dizziness, or imbalance^21^	Area under the curve for a serious diagnosis (stroke, TIA, vertebral artery dissection, tumor) was 0.95, sensitivity 100% and specificity 69.2% (for score <5)	Requires accurate BPPV assessment; further prospective multicenter validation is needed before routine use.
Tools to diagnose PCS using physical examination findings						
HINTS^22^	Single-center study of 101 patients with acute vestibular syndrome	To distinguish central (usually stroke) vs. peripheral (usually neuritis) in AVS patients	Head impulse, nystagmus, test of skew	Yes: systematic review and meta-analysis of studies evaluating HINTS in neurologists and emergency medicine physicians^23^	Performed by neurologists (neuro-otology or vascular neurology), sensitivity and specificity were 94.3% / 97.6%. Performed by any physician (neuro or EM): 95% / 89.1%	High accuracy only in appropriately selected patients with AVS and spontaneous nystagmus when performed by trained clinicians; not a universal dizziness screen.
STANDING^24^	Single-center study of 292 ED patients with vertigo	To differentiate central from noncentral acute vertigo	SponTAneous Nystagmus, Direction, head Impulse test, and standiNG	Yes: systematic review/ meta-analysis evaluating ability of EM physicians to perform STANDING^25^	Performed by EM physicians, STANDING had sensitivity of 0.96 and specificity of 0.88	Promising when performed by trained emergency physicians, but requires correct patient selection and broader multicenter validation.

ASPOS, Adam’s Scale of Posterior Stroke; ED, emergency department; EM, emergency medicine; e-NIHSS, expanded NIHSS; HINTS, Head Impulse, Nystagmus, and Test of Skew; IVBSS, Israeli Vertebrobasilar Stroke Scale; mRS, modified Rankin Score; NIHSS, NIH stroke scale; PCS, posterior circulation stroke; POST-NIHSS, Posterior NIHSS; BATMAN, Basilar Artery Treatment and Management registry (sites in Australia, New Zealand, Europe, and United States); TriAGe+, No Triggers, Atrial fibrillation, Gender, Blood pressure, Brainstem or cerebellar dysfunction, clinical features (focal weakness or speech impairment), Dizziness (not = vertigo), no history of vertigo or dizziness of labyrinth or vestibular disease.

### 2.2 Tools to Diagnose PCS Using History and Clinical Features

Diagnostic scores based on history and symptom profiles have also been evaluated. The TriAGe+ score was derived in a single-center cohort of 498 ED patients with dizziness or vertigo and subsequently prospectively validated in a separate single-center cohort of 357 patients.^18^^,^^19^ In the validation study, TriAGe+ had higher specificity than the Head Impulse Test (HI-) Characterization of Spontaneous Nystagmus (-N-) Test of Skew (-TS) (HINTS) performed by trained physicians for identifying central causes of dizziness (96.3% vs 85.9%). However, its weighting favors “dizziness” over “vertigo” and relies on symptom quality, which does not meaningfully improve diagnostic accuracy in suspected PCS.^6^ The Sudbury Vertigo Score was developed prospectively in 2078 patients across 3 EDs and subsequently validated in a separate retrospective cohort of 4559 ED patients.^21^^,^^26^ It demonstrated 100% sensitivity and 72.1% specificity for serious diagnoses at a score below 5, but it relies in part on an accurate diagnosis of benign paroxysmal positional vertigo (BPPV).^27^ Neither score has been prospectively validated across multiple centers in nonexpert clinicians or compared directly with all other available approaches. They should, therefore, be considered investigational adjuncts rather than standalone tools for routine ED decision making.

### 2.3 Tools to Diagnose PCS Using Physical Examination Findings

Physical examination-based approaches are most useful when matched to the appropriate dizziness syndrome. In patients with acute vestibular syndrome (AVS) and spontaneous nystagmus, the HINTS examination can distinguish central from peripheral causes with high accuracy when performed by trained clinicians (sensitivity 96.7%, specificity 94.8%).^23^^,^^26^ Its performance is substantially lower without formal training, and inappropriate use in patients without continuous symptoms or nystagmus limits its value in routine practice. A 2025 survey reported that fewer than 30% of emergency physicians routinely use HINTS, with most citing inadequate training and low confidence in interpretation.^28^ In another study, only 18% felt adequately trained, and fewer than 10% reported using it regularly.^29^ The four-step bedside diagnostic algorithm (STANDING) protocol, which also incorporates positional maneuvers and gait assessment, has shown similar accuracy when performed by trained emergency physicians (sensitivity 96%, specificity 88%).^25^ These results come largely from selected populations and trained examiners, and head-to-head, multicenter comparisons are lacking. HINTS should, therefore, be reserved for patients with AVS and spontaneous nystagmus when the clinician is trained in its performance and interpretation; gait unsteadiness and a focused neurologic examination are more informative when nystagmus is absent.^30^ Nystagmus is absent in many PCS presentations,^31^ and only a small minority of all ED dizziness presentations meet the selected criteria used in AVS studies;^3^^,^^22^^,^^30^ neither HINTS nor STANDING is a universal screening examination.

### 2.4 Imaging Considerations

For identification of acute small-vessel PCS, magnetic resonance imaging (MRI) with diffusion-weighted imaging (DWI) is more sensitive than noncontrast head CT; one study reported sensitivities of 95% and 41.8%, respectively, at approximately 6 hours.^32^, ^33^, ^34^ However, rapid MRI is not continuously available in many hospitals, and early DWI may be negative in acute ischemic stroke. A meta-analysis found a 6.8% prevalence of DWI-negative acute ischemic stroke, with posterior circulation ischemia associated with fivefold higher odds of a negative scan.^35^ When early MRI is negative but clinical concern persists, the reference standard is often the subsequent clinical course, repeat MRI, or additional vascular imaging rather than the initial scan alone. Imaging should, therefore, be selected according to the clinical syndrome and local resources: MRI is appropriate when bedside findings are central or equivocal, whereas computed tomography angiography (CTA) or magnetic resonance angiography (MRA) should be considered when transient ischemic attack or large-vessel occlusion is suspected.^30^

Evidence for thrombolysis in patients with isolated dizziness or imbalance and for reperfusion in isolated cerebellar infarction remains limited and largely observational outside basilar artery occlusion.^5^^,^^36^ Observational and rehabilitation data also describe substantial functional recovery after cerebellar stroke, potentially reflecting cerebellar compensation.^37^^,^^38^ These considerations caution against applying the anterior circulation treatment paradigm uniformly to all PCS, although prompt recognition remains essential for suspected basilar artery occlusion and for other patients who may be eligible for reperfusion therapy.

## 3 Practical Application in the ED

Most ED patients with isolated dizziness do not have stroke. In a recent cohort of 77,315 patients discharged after an ED dizziness visit, the 30-day incidence of stroke hospitalization was 0.12%, and stroke with disability or mortality occurred in 0.04%; 59% of subsequent infarcts were in the anterior circulation and may not have been related to the index presentation.^39^ This low individual risk should temper criticism of emergency physicians and underscores the unfavorable signal-to-noise ratio of broadly imaging all patients with dizziness. The goal is not universal stroke testing, but accurate syndrome classification and recognition of focal or high-risk features.

Practically, clinicians should first distinguish triggered episodic vestibular syndrome from spontaneous episodic or continuous AVS based on timing and triggers rather than symptom labels alone.^30^ In patients with triggered positional episodes and no spontaneous nystagmus, the Dix-Hallpike test should be used to identify posterior-canal BPPV. A positive test (transient symptoms with up-beating torsional nystagmus) in an otherwise neurologically intact patient definitively diagnoses BPPV. Atypical findings or persistent concern warrant further evaluation. In AVS with spontaneous nystagmus, trained clinicians may use HINTS or STANDING; central or equivocal findings should prompt stroke-protocol MRI and specialist consultation. In patients without nystagmus, HINTS is not applicable. A focused neurologic examination, including gait and truncal stability, is more appropriate. Sudden diplopia, dysarthria, dysphagia, focal limb or truncal ataxia, new unilateral weakness or numbness, severe gait impairment, or altered consciousness should prompt urgent evaluation for stroke. Sudden unexplained coma or alterations in consciousness (which never occur with peripheral causes of dizziness) should prompt urgent vascular imaging for possible basilar artery occlusion.^5^

Education and systems interventions should support, rather than substitute for, clinical judgment. Only 17.4% of emergency medicine residency programs surveyed in 2005 required a neurology rotation and 15.2% required neurosurgery, illustrating longstanding variability in formal neurologic training.^40^ More recent surveys also identify limited HINTS training and confidence.^28^^,^^29^^,^^41^ Programs should provide repeated, competency-based instruction in syndrome classification, Dix-Hallpike and Epley maneuvers, HINTS/STANDING patient selection and interpretation, and gait assessment. ED protocols should clarify when bedside testing, MRI, vascular imaging, or neurology consultation is appropriate and should include pathways for patients with persistent clinical concern despite nondiagnostic initial imaging. Prospective, multicenter studies are needed to compare these approaches in nonexpert clinicians and determine which interventions improve patient-centered outcomes.

## 4 Conclusion

PCS among patients with dizziness is a clinically important but uncommon diagnosis, and no single bedside examination, clinical score, or imaging strategy has been shown to be superior across undifferentiated, multicenter ED populations. Existing tools should be applied according to the clinical syndrome and the clinician's training rather than adopted as universal screening tests. Emergency physicians are generally working within the limitations of subtle presentations, imperfect reference standards, variable access to MRI, and incompletely validated tools, some of which (HINTS and STANDING) are not part of residency curricula.

At present, the most practical approach is to classify dizziness by timing and triggers; perform a focused neurologic examination with gait assessment; use Dix-Hallpike testing for triggered positional symptoms; reserve HINTS or STANDING for appropriately selected patients with spontaneous nystagmus and trained examiners; and obtain MRI, vascular imaging, or neurologic consultation when bedside findings are central, equivocal, or discordant with the clinical course. Systems should support this approach through competency-based education, workup and consultation pathways, and access to repeat evaluation when early testing is nondiagnostic. Future multicenter comparisons should determine which combinations of bedside examination, imaging, and decision support improve patient-centered outcomes.

## Author Contributions

All authors contributed to the conception and design of the manuscript, critically reviewed and revised the manuscript for important intellectual content, and approved the final version. Drs. Klein and Seiffert contributed to the initial manuscript draft. Drs. Mayes and Edlow provided overall project oversight.

## Funding and Support

By *JACEP Open* policy, all authors are required to disclose any and all commercial, financial, and other relationships in any way related to the subject of this article as per ICMJE conflict of interest guidelines (see www.icmje.org). The authors have stated that no such relationships exist.

## Conflicts of Interest

Dr. Adeoye reports research and salary support from the National Institutes of Health/National Institute of Neurological Disorders and Stroke (NIH/NINDS). He is also a founder and equity holder in Sense Diagnostics, Inc. Dr. Moore reports receiving research funding from Philips/BioTel. Dr. Edlow reports serving as a reviewer of medical malpractice cases for both defense and plaintiff firms, some of which involve patients with posterior circulation stroke. He also served as chairperson of the Society for Academic Emergency Medicine (SAEM) GRACE-3 committee, which produced a clinical guideline on the management of patients with dizziness. Dr. Mayes reports research and salary support from the National Institutes of Health/National Institute of Neurological Disorders and Stroke (NIH/NINDS). The rest of the authors have affirmed they have no conflicts of interest to declare.
